# Supplementary material for: Src-FAK Signaling Mediates Interleukin 6-Induced HCT116 Colorectal Cancer Epithelial–Mesenchymal Transition
Source: Int J Mol Sci. 2023 Apr 2;24(7):6650. doi: 10.3390/ijms24076650 (PMC10095449; doi:10.3390/ijms24076650)
Supplement: Supplementary file 1 [file ijms-24-06650-s001.zip › ijms-2285780-Supplementary.pdf]

Supplement Information

Supplement Methods

Cell viability assay

Cell viability was measured by the colorimetric 3-(4,5-dimethylthiazol-2-yl)-2,5-diphenyl tetrazolium bromide (MTT) assay. Cells (2×10^4 cells/well) were seeded in 48-well plates. After various treatments, 1 mg/ml MTT was added to the culture plates and incubated at 37 °C for an additional 1 h. Cells were lysed in 300 µl dimethyl sulfoxide (DMSO). The absorbance at 550 nm was measured on a microplate reader. Each experiment was performed in triplicate. To reveal relevant trends and to control for unwanted sources of variation, normalization was performed to compare the differences after the treatment. The viability of cells in control group was considered to be 100 % and the viability of the cells treated with indicated inhibitors was expressed as a percentage of the control.

Supplement Figures

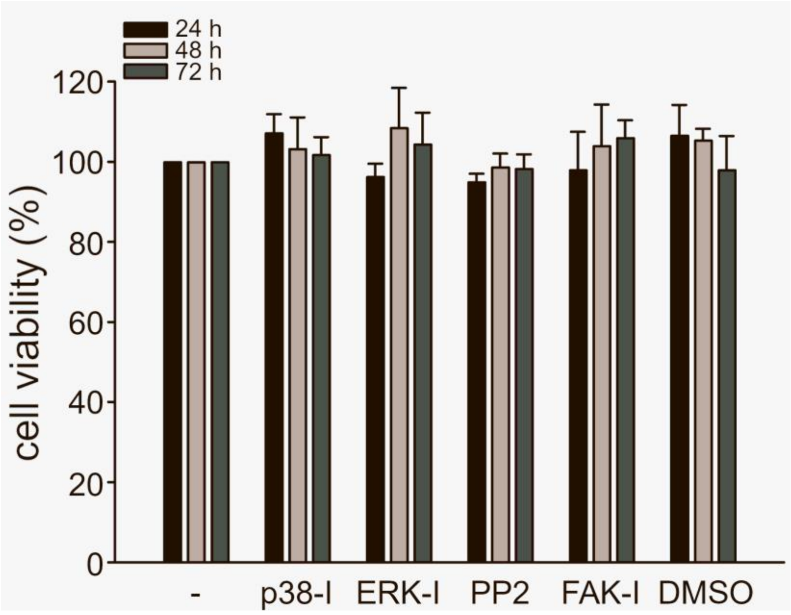

Supplement Figure S1. Effects of inhibitors on cell viability in HCT116 colorectal cancer cells. HCT116 cells were treated with 3 μM p38 MAPK inhibitor III (p38-I), 3 μM ERK activation inhibitor peptide I (ERK-I), 1 μM PP2, 1 μM NSC 667249 (FAK inhibitor, FAK-I) or 0.1 % DMSO for indicated time periods. After treatment, cell viability was determined by an MTT assay. Each column represents the mean ± S.E.M. of five independent experiments performed in triplicate.

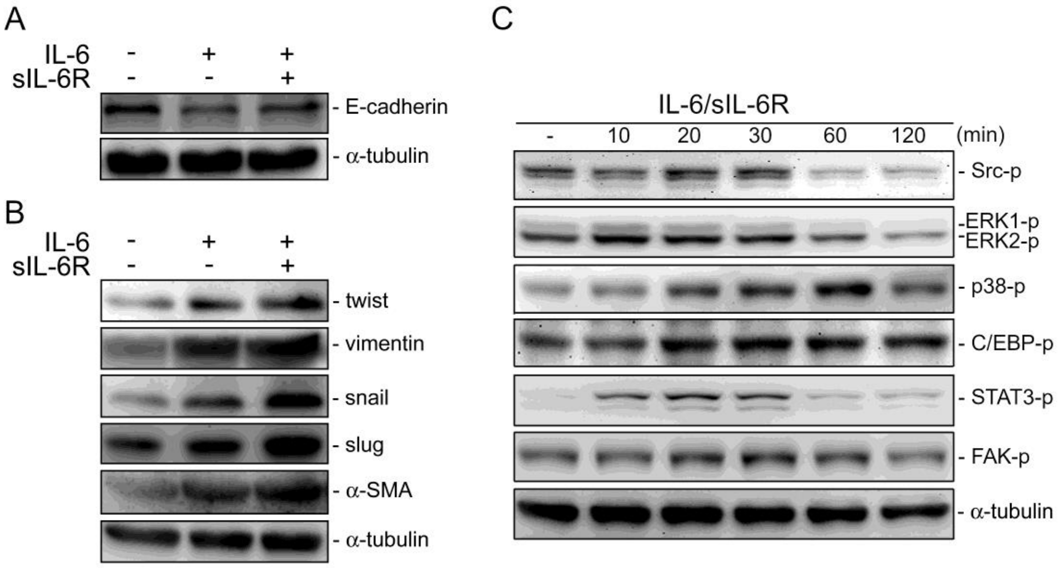

Supplement Figure S2. Effects of IL-6 on epithelial and mesenchymal markers in HT-29 colorectal cancer cells. HT-29 Cells were treated with vehicle, IL-6 (20 ng/ml) or IL-6 plus sIL6R (20 ng/ml) for 72 (A) or 48 (B) h. After treatment, cells were harvested for assessing the extent of E-cadherin (A), twist, vimentin, snail, slug or α-SMA was determined by immunoblotting (B). (C) HT29 cells were treated with IL-6 (20 ng/ml) plus sIL6R (20 ng/ml) for indicated time periods. The phosphorylation status of Src, ERK1/2, p38MAPK, C/EBP, STAT3 or FAK was determined by immunoblotting.
